# Successful isolation of viable stem cells from cryopreserved microfragmented human adipose tissue from patients with knee osteoarthritis – a comparative study of isolation by tissue explant culture and enzymatic digestion

**DOI:** 10.1186/s40634-023-00596-x

**Published:** 2023-03-23

**Authors:** Jasmin Bagge, Per Hölmich, Freja Aabæk Hammer, Jan O. Nehlin, Kilian Vomstein, Lars Blønd, Lisbet Rosenkrantz Hölmich, Kristoffer Weisskirchner Barfod

**Affiliations:** 1 Sports Orthopedic Research Center – Copenhagen (SORC-C), Department of Orthopedic Surgery, Copenhagen University Hospital – Amager and Hvidovre, Kettegård Allé 30, 2650 Hvidovre, Denmark; 2 Department of Clinical Research, Copenhagen University Hospital – Amager and Hvidovre, Kettegård Allé 30, 2650 Hvidovre, Denmark; 3 Department of Obstetrics and Gynecology, The Fertility Clinic, Copenhagen University Hospital – Hvidovre, Kettegård Allé 30, 2650 Hvidovre, Denmark; 4 Department of Orthopedic Surgery, Zealand University Hospital – Køge, Lykkebækvej 1, 4600 Køge, Denmark; 5 Department of Plastic Surgery, Copenhagen University Hospital – Herlev and Gentofte, Borgmester Ib Juuls Vej 1, 2730 Herlev, Denmark

**Keywords:** Stem cells, Cryopreservation, Biobanking, Adipose tissue, Microfragmentation, Enzymatic digestion, Tissue explant culture

## Abstract

**Purpose:**

To investigate if viable stem cells could be isolated and expanded from cryopreserved microfragmented adipose tissue (AT) harvested from patients with knee osteoarthritis.

**Methods:**

Microfragmented abdominal AT from knee osteoarthritis patients was cryopreserved at -80 °C in cryoprotectant-medium. The samples were thawed for stem cell isolation by tissue explant culture (TEC) and enzymatic digestion (ED), respectively. Viability, population doublings, and doubling time were assessed by trypan blue staining and flow cytometry. Cell type and senescence-associated β-galactosidase activity were analyzed by flow cytometry. Osteogenic and adipogenic differentiation was assessed quantitatively by Alizarin-Red-S and Oil-Red-O staining, respectively.

**Results:**

Microfragmented AT from 7 patients was cryopreserved for a period of 46–150 days (mean (SD) 115.9 days (44.3 days)). Viable stem cells were successfully recovered and expanded from all patients using both isolation methods with no significant difference in viable population doublings or doubling time from passage 1 to 3 (*p* > 0.05). Low levels of senescence-associated β-galactosidase activity was detected for both methods with no significant difference between TEC and ED (*p* = 0.17). Stemness was verified by stem cell surface markers and osteogenic and adipogenic differentiation performance. Adventitial stem cells (CD31^−^CD34^+^CD45^−^CD90^+^CD146^−^), pericytes (CD31^−^CD34^−^CD45^−^CD90^+^CD146^+^), transitional pericytes (CD31^−^CD34^+^CD45^−^CD90^+^CD146^+^), and CD271^+^ stem cells (CD31^−^CD45^−^CD90^+^CD271^+^) were identified using both methods. More pericytes were present when using TEC (25% (24%)) compared to ED (3% (2%)) at passage 4 (*p* = 0.04).

**Conclusions:**

Viable stem cells can be isolated and expanded from cryopreserved microfragmented AT using both TEC and ED. TEC provides more clinically relevant pericytes than ED.

## Background

Osteoarthritis is an inflammatory and degenerative joint disease, which causes cartilage break-down and damage to the subchondral bone. Osteoarthritis is a major health problem affecting more than 300 million people globally [1]. Over the past two decades, intraarticular treatment of osteoarthritis with different types of stem cells has shown promising results in regard to improved joint healing and pain relief [2–6]. Adipose tissue (AT) is a rich source of multipotent stem cells and several subtypes have been identified based on their surface markers. In the perivascular niche, adventitial stem cells (ASCs) (CD31^−^CD45^−^CD34^+^CD90^+^CD146^−^) and pericytes (CD31^−^CD45^−^CD34^−^CD90^+^CD146^+^) have been identified. In the stromal vascular fraction, mesenchymal stromal cells (MSCs) (CD34^−^CD45^−^CD146^−^CD90^+^CD105^+^) have been identified [7]. Moreover, a subpopulation of CD271^+^ stem cells have been identified in the stromal vascular fraction with higher osteogenic potential than CD271^−^ cells [8]. AT-derived stem cells have thus gained high clinical interest as a regenerative treatment of osteoarthritis due to their abundance and ease of harvest by lipoaspirates [2].

Routine processing of whole lipoaspirates for therapeutic use consists of enzymatic digestion and in vitro stem cell expansion prior to injection. Complex regulatory issues related to standard application of enzymatically treated and expanded cells have led to the development of mechanical microfragmentation of AT to harvest stem cells ready for treatment in a one-step surgical procedure [9]. Treatment of osteoarthritis with stem cells from autologous microfragmented AT has proven safe (approved by the FDA in 2016) and has shown positive effects on knee osteoarthritis, measured by an improved Knee Injury and Osteoarthritis Outcome Score (KOOS) and Visual Analog Scale (VAS) in humans [10, 11], and decreased synovitis and improved cartilage healing in a rabbit model [2]. Microfragmentation of AT is used increasingly in the clinic as a stem cell therapy for knee osteoarthritis, even though there are no randomized controlled trials proving efficacy and efficiency of the treatment [12].

Cryopreservation and biobanking of stem cells are important for research purposes and for treatment of aged patients, as aging has demonstrated negative effects on the potential of AT-derived stem cells when treating osteoarthritis [13–16]. Cryopreservation potentially opens the possibility to store functional stem cells from an early age for use later in life. Moreover, cryopreservation of microfragmented AT would allow for repeated stem cell treatments, which have shown improved long-term results in clinical trials [3, 6], without the need for additional liposuctions.

A limited number of human studies have described successful isolation of stem cells from cryopreserved whole lipoaspirates [17–19], but there is no methodology study investigating isolation of stem cells from cryopreserved microfragmented AT.

The aim of this study was, therefore, to investigate if viable stem cells could be isolated and expanded from cryopreserved microfragmented subcutaneous abdominal AT by two different isolation methods: (1) tissue explant culture and (2) enzymatic digestion.

## Methods

The study was a comparative methodology study performed at Department of Orthopedic Surgery and Department of Clinical Research at Copenhagen University Hospital – Hvidovre, Denmark in the period from November 2021 to October 2022 with harvesting of AT from November 2021 to February 2022. Collection, molecular analysis, and biobanking of the cells were approved by the Danish National Committee on Health Research Ethics (H-18013145) and the Danish Data Protection Agency (VD-2018–141).

### Study population

Patients were included from an ongoing randomized controlled trial investigating treatment of knee osteoarthritis with autologous microfragmented AT (ClinicalTrials.gov Identifier: NCT03771989) [20]. Inclusion criteria were defined as patients aged 18 to 70 years suffering from pain and functional impairment due to osteoarthritis Kellgren-Lawrence grades 2–3 in the tibiofemoral joint [20]. The size of the study population was determined by logistical factors. For a period of 4 months from November 2021 to February 2022 no immediate processing of the harvested microfragmented AT was possible, due to logistic and stem cell research personnel reasons. The samples were thus cryopreserved for later stem cell isolation and analysis.

### Adipose tissue collection

Subcutaneous abdominal AT was harvested under local analgesia and sterile conditions as described previously [10, 20]. The patient was positioned supine and the harvest performed through two stab incisions in the area between the umbilicus and the pubic bone. The subcutaneous AT was prepared for harvesting by injection of a suspension of 250 mL isotonic saline with 400 mg lidocaine, 0.4 mg adrenaline, and 10 mmol bicarbonate. 80–100 mL AT was harvested by liposuction using a 13G blunt cannula connected to a Vaclock® 20 mL syringe. Using sedimentation, excess saline suspension was separated from the AT and removed from the syringe. After the procedure, the skin was closed with a band aid. An elastic compression bandage was given to the patient to be used for 3–4 weeks post liposuction.

### Microfragmentation of adipose tissue

In the operation theater, the harvested AT was immediately microfragmented using a Lipogems® processing kit (Lipogems, Milano, Italy, Cat#LG-SK-240) as per manufacturer’s instructions. Isotonic saline was added to the device containing 5 stainless steel marbles and the AT clusters were progressively reduced in size by mechanical shaking to release stem cells, while washing out blood residues [9]. Microfragmented AT was collected in two 10 mL syringes. Excess saline suspension was separated from the AT and removed from the syringes by means of sedimentation.

### Cryopreservation of microfragmented adipose tissue

In the operation theater, 3 mL of microfragmented AT was transferred to a 50 mL conical tube (TPP, Cat#91,050) containing 10 mL ice-cold, sterile-filtered (0.2 µm, SFCA sterile filter, Thermo Scientific, Cat#723–2520) cryomedium consisting of Fetal Bovine Serum (8.7 mL FBS, Gibco, Cat#10,270–106) and a final concentration of 10% dimethyl-sulfoxide (%V/V) (1.3 mL DMSO, Sigma-Aldrich, Cat#D2650-100ML). The tube was inverted and immediately transported on ice to the hospital freezer facility, where it was transferred to a Styrofoam box for slow freezing and storage at -80 °C.

### Thawing and isolation of adipose tissue-derived stem cells

Standard methods for isolating and purifying AT-derived stem cells from fresh and cryopreserved whole AT was modified and applied using both tissue explant culture (TEC) and enzymatic digestion (ED) [9, 17, 18]. TEC represent non-enzymatically treated cells embodying fresh stem cells from microfragmented AT used for the treatment of osteoarthritis. ED represents a well-known method to isolate stem cells from the stromal vascular fraction.

For thawing, the sample was transported on ice to the laboratory. The tube was immediately transferred to a 37 °C water bath until complete thawing (approximately 5 min), cleaned with 70% ethanol, and transferred to a laminar air flow bench. The cryomedium was removed by slowly decanting the sample to a 40 µm cell strainer (Falcon, Cat#352,340) attached to a 50 mL conical tube, where the microfragmented AT was kept in the cell strainer. The sample was washed 3 × with 5 mL pre-warmed (37 °C) Dulbecco’s phosphate buffered saline (dPBS, Gibco, Cat#14,190–144) while kept in the cell strainer to remove left-over DMSO. Using a sterile anatomical forceps, microfragmented AT was divided for stem cell isolation and culture by TEC and ED, respectively.

### Isolation by tissue explant culture

Microfragmented AT was transferred to two T75 cm^2^ culture flasks (TPP, Cat#90,076) with 1 mL microfragmented AT per T75 cm^2^ flask for tissue explant culture. 11 mL pre-warmed (37 °C) expansion media was added per T75 cm^2^ flask consisting of Dulbecco’s modified Eagle’s medium (DMEM, 1 g/L glucose, with phenol red, GlutaMAX, and pyruvate, Gibco, Cat#10,567–022), 10% (v/v) FBS, and 1% (v/v) penicillin/streptomycin (P/S, Gibco, Cat#15,070,063). After 48 h, AT clusters and non-adherent cells were aspirated along with the expansion medium and washed with 6 mL dPBS/T75 cm^2^ flask. The cells were cultured at 37 °C in a humidified atmosphere containing 5% CO_2_. Expansion medium was changed every 2–3 days.

### Isolation by enzymatic digestion

1 mL microfragmented AT was transferred to a 50 mL conical tube with 10 mL sterile filtered (0.2 µm) enzyme medium consisting of DMEM, 1% (v/v) P/S, and 1 mg/mL collagenase type I (Gibco, Cat#17,018–029). The tissue was enzymatically digested by incubation at 37 °C and 30 rpm for 45 min. The enzyme medium containing the released cells was transferred through a 100 µm cell strainer (Corning, Cat#431,752), and the pellet with cells was washed twice in sterile dPBS and centrifuged at 500 × g for 5 min between the washes.

A small aliquot was used for cell counting and cellular viability (of all nucleated cells) by the exclusion of trypan blue stain (Fisher Scientific, Gibco, Cat# 11,538,886) when using a Fast-Read® 102 (VWR, Cat#630–1893). To further assess viability of the stem cells immediately after thawing, a quantitative LIVE/DEAD® Viability/Cytotoxicity Kit for mammalian cells (Invitrogen, ThermoFisher Scientific, Cat# L3224) was used as per manufacturer’s protocol using flow cytometry. In short, 0.5 × 10^6^ cells were stained with 50 µM calcein AM and 2 mM ethidium homodimer-1 for 20 min protected from light. The cells were analyzed with a BD LSR Fortessa flow cytometer with FACSDiva 8.0.3 software using a FITC filter for calcein AM and a PE-Texas Red filter for ethidium homodimer-1. The cells were gated on to exclude debris and leucocytes by size and granulation.

The pellet was re-suspended in 11 mL expansion medium and distributed into 1 T75 cm^2^ flask. The first medium change occurred after 48 h. In accordance with accepted AT-derived stem cell isolation protocols, viable and adherent cells were considered to represent AT-derived stem cells 48 h after plating [18]. The cells were cultured at 37 °C in a humidified atmosphere containing 5% CO_2_. Expansion medium was changed every 2–3 days.

### Cell expansion

At approximately 70% confluence, the cells were passaged with 1.5 mL 0.25% Trypsin/1 mM EDTA (Gibco, Cat# 11,560,626) per T75 cm^2^ culture flask. Cell counting and assessment of cellular viability was performed as described above. The cells were grown with a seeding density of 500,000 cells per T75 cm^2^ flask. The cells were grown in expansion medium at 37 °C and 5% CO_2_ with medium change 2–3 times per week.

### Population doublings and doubling time

To assess proliferative potential, the enzymatically digested cells were counted immediately after thawing and when reaching approximately 70% confluence as described above. Due to the physical consistency of microfragmented AT grown as tissue explants without enzymatic treatment, counting of cells immediately after thawing was not possible. All cells were counted with trypan blue at passage 1, 2, and 3. The cumulative population doublings (cPDs) and doubling time (DT) was calculated using the following formulas [17]:

$$cPDs=Log \left(\frac{N}{N0}\right)* 3.33$$

$$DT=\left(\frac{CT}{cPDs}\right)$$

where N0 is the initial number of live cells plated, N is the number of live cells harvested at ~ 70% confluence, and CT is the total time in days cultured.

### Immunophenotyping by flow cytometry

At passage 4, adherent cells underwent immunophenotype analysis using flow cytometry. The cells were trypsinized, counted, and washed twice in dPBS. The cells were then resuspended in FACS buffer (Low glucose DMEM without phenol red (Gibco, Fisher Scientific, Cat# 11,580,406) and 10% FBS) with 0.5 × 10^6^ cells per FACS tube (Fisher Scientific, Cat# 10,585,801). The cells were tested in duplicates. To minimize staining artifacts when using multiple fluorophores, BD Horizon Brilliant Stain Buffer Plus (BD Biosciences, Cat# 566,385) was added to the tubes as per manufacturers’ instructions. To identify the specific stem cell subpopulations, the cells were analyzed with multiple anti-human fluorescent primary antibodies; CD31-FITC, CD34-APC, CD45-BV786, CD90-PE, CD146-BV421, and CD271-PE-Cy^TM^7 (BD Biosciences, Horizon subtype, mouse IgG_1_κ isotype) in a 1:100 dilution. The cells were incubated for 20 min at room temperature in the dark according to manufacturers’ instructions. Thereafter, the cells were washed twice with 1 mL FACS buffer to remove non-adherent antibodies. A third aliquot of the cells was processed in the same way but without primary antibodies and used as a non-stained negative control for each cell line. Each pellet was resuspended in 350 µL FACS buffer for immediate analysis using a flow cytometer (BD LSR Fortessa with BD FACSDiva 8.0.3 software) recording 30,000 events. The cells were gated on to exclude debris and singlet cells were selected. Next the cells were gated to identify the CD90^+^, CD45^−^, and CD31^−^ fraction, and lastly for the different subtypes. BD compensation beads (BD CompBeads with anti-mouse IgGκ) and single-color-stained cells were used to perform compensation on the flow cytometer and to account for autofluorescence. Non-stained cells, fluorescence-minus-one (FMO), and mouse IgG_1_κ isotype controls for each fluorophore (BD bioscience) were used to optimize accurate gating and to test the protocol. Performance of the flow cytometer was checked prior to each assay using BD FACSDiva™ CS&T Research Beads (BD Biosciences) in FACS buffer. Analysis of flow cytometry data was performed using BD FACSDiva Software 8.0.3.

### Osteogenic and adipogenic in vitro differentiation

Passage 3 cells were trypsinized, counted, and seeded as passage 4 monolayer cells with a seeding density of 3000 cells/cm^2^ in a 6-well plate (NUNC, Fisher Scientific, Cat# 10,469,282). The cells were initially grown in expansion medium at 37 °C and 5% CO_2_ with medium change every 2–3 days. Upon ~ 90% confluence, expansion medium was removed and osteogenic and adipogenic differentiation was induced, respectively. Osteogenic induction medium consisted of DMEM (1 g/L glucose), 10% FBS, 1% P/S, 100 nM dexamethasone (Sigma Aldrich, Cat# D4902), 0.05 mM L-ascorbic acid-2-phosphate sesquimagnesium salt hydrate (Sigma Aldrich, Cat# A8960), and 10 mM β-glycerophosphate disodium salt hydrate (Sigma Aldrich, Cat# G9422) as previously described [21, 22]. Adipogenic induction medium consisted of DMEM (1 g/L glucose), 10% FBS, 1 µM dexamethasone, 10 µg/mL insulin (Sigma Aldrich, Cat# 11,376,497,001), 500 µM 3-iso-butyl-1-methylxanthine (Sigma Aldrich, Cat# 15,879), and 200 µM indomethacin (Sigma Aldrich, Cat# 17,378) as previously described [22]. Cells were kept under adipogenic induction medium for 14 days and osteogenic induction medium for 21 days, respectively, with medium change every 3 days. All samples were run in triplicates, with an equal number of non-induced cells kept under normal expansion medium throughout the entire assay as a negative control.

### Oil-Red-O staining

After 14 days of adipogenic induction, adipogenic differentiation performance was evaluated quantitatively by Oil-Red-O staining to determine cellular accumulation of lipid vacuoles using a modified protocol based on Kraus et al*.* [23, 24]. In short, the cells were washed once with dPBS and fixed with 4% formaldehyde (methanol-free, Fisher Scientific, Cat# 11,586,711) in dPBS for 15 min at room temperature. The formaldehyde was removed, and the cells were stained with 1.25 mL/well 0.2% Oil-Red-O solution (Sigma-Aldrich, Cat# O0625) in 40% 2-propanol (Sigma-Aldrich, Cat# I9516) and incubated for 30 min at room temperature on an orbital shaker. The work solution was made fresh and filtered once before every use. The cells were then washed 5 times with distilled water to remove excess dye. To elute the dye, 2 mL/well 100% 2-propanol was added and incubated for 10 min at room temperature on an orbital shaker. 2 × 200 µL of the elute was transferred to a 96-well plate (clear, flat bottom, NUNC, Sigma-Aldrich, Cat# P7366-1CS) for duplicate testing. A duplicate of 40% 2-propanol in distilled water was used as a blank control. A standard curve of Oil-Red-O solution from 0.2% to 0.00625% was made through serial-dilutions and tested in duplicates. Absorbance was measured with a microplate reader (ELX808, Bio-Tek Instruments) at 490 nm. For calculations, the duplicates were averaged and the blank subtracted. The standard curve was plotted to determine a linear equation and trendline. Oil-Red-O concentration was determined according to the equation of the trend line.

For histological visualization, one cell replicate per isolation method (n = 2) were counterstained with 2 mL/well Mayer’s hematoxylin solution (Sigma Aldrich, Cat# MHS16) for 2 min at room temperature after Oil-Red-O staining (not treated with 2-propanol to elude the dye). To remove excess counterstain, the cells were washed 3 times with distilled water. Lipid vacuoles were visualized at 20X under phase contrast microscopy (Zeiss Axio Vert.A1) of 3 non-overlapping fields per well.

### Alizarin Red S assay

After 21 days of osteogenic induction, calcium deposition was measured quantically with a colorimetric Alizarin Red S (ARS) quantification kit (ARed-Q, ScienCell, Cat# 8678) following manufacturers protocol based on Gregory et al*.* [25]. In short, the cells were gently washed 3 times in dPBS without calcium and magnesium (Gibco, Fisher Scientific, Cat# 14,190,144) before being fixed in 4% formaldehyde for 15 min at room temperature. The fixative was removed and the cells washed 3 times with milli-Q water. Calcium deposits were then visualized by adding 40 mM ARS staining for 25 min with gentle shaking. Next, the cells were washed 5 times with milli-Q water to remove excess dye. For histological visualization, calcium deposits from one cell replicate per isolation method (*n* = 2) were visualized by phase contrast imaging at 20 × magnification with a Zeiss Axio Vert.A1 microscope of 3 non-overlapping fields per well. Calcified minerals were extracted at low pH by adding 10% acetic acid for 30 min with shaking and a cell-scraper, followed by a heating process in parafilm-coated microcentrifuge tubes at 85 °C for 10 min and a cooling process on ice for 5 min. The slurry was centrifuged at 20,000 × g for 15 min. Finally, the solution was neutralized with 10% ammonium hydroxide (pH between 4.1 and 4.5). ARS concentration was quantified by colorimetric detection at 405 nm in a flat-bottomed 96-well plate using a microplate reader (ELX808, Bio-Tek Instruments). ARS concentration of the samples was calculated based on a standard curve incorporated in the assay and by subtracting the mean blank value from all samples. All technical replicates were analyzed in triplicates with 3 osteogenic-induced and 3 non-induced wells per cell line. ARS concentration was averaged per cell line per isolation method.

### Cellular senescence

Cellular senescence was assessed on adherent cells at passage 4 using a quantitative senescence-associated β-Galactosidase (SA-BGAL) activity assay kit for flow cytometry modified to manufacturer’s protocol (Abcam, Cat# ab228562). The cells were seeded in 24-wells with a seeding density of 1 × 10^5^ cells per well and grown in expansion medium for 48 h at 37 °C and 5% CO_2_ to allow for plastic-adherence. The medium was then replaced with expansion medium containing 1.5 µL of senescent dye per 500 µL medium and incubated for 2 h in the dark at 37 °C and 5% CO_2_. The senescence dye contains a green fluorogenic SA-BGAL substrate that enters live cells where it gets cleaved by beta-galactosidase, which generates a strong green fluorescence. The cells were tested in duplicates. The medium was removed, and the cells washed twice with wash buffer (pH 6) from the kit. Next, the cells were harvested by trypsinization (0.25% Trypsin–EDTA, Gibco, Thermo Fisher) and transferred to a FACS tube where they were washed twice with wash buffer (pH 6). The samples were resuspended in 350 µL wash buffer for analysis and protected from light. SA-BGAL activity green fluorescence (excitation: 490 nm, emission: 514 nm) was detected immediately with a flow cytometer (BD LSR Fortessa with BD FACSDiva 8.0.3 software) using a FITC channel and recording between 10–30,000 events. Non-stained cells were used as a control for each cell line. The cells were gated to exclude debris. A side scatter (SSC) versus FITC plot was used to gate on cells. A positive senescent control that consisted of microfragmented AT-derived stem cells, serially cultured in vitro at low seeding densities for 7 months, until reaching replicative senescence (denoted by the absence of apparent proliferation after 2–3 weeks) were used to optimize gating and account for autofluorescence due to lipofuscin accumulation, which is a hallmark of cellular senescence [26, 27]. Analysis of flow cytometry data was performed related to the positive control gating using BD FACSDiva Software 8.0.3. The results from the stained sample duplicates were averaged for further analysis.

### Statistical analysis

Comparison between TEC and ED was performed using paired t-tests. Normality of data was confirmed using Shapiro–Wilk tests and QQ-plots. *p*-values < 0.05 were considered statistically significant. GraphPad Prism (version 9.3.1) was used for all statistical analysis. Due to low number of observations, it was chosen not to perform any further statistical correlative analysis.

## Results

Microfragmented AT from 7 (5 female and 2 male) knee osteoarthritis patients with an age between 41 and 63 years (mean 52.6 years, SD 8.1 years) were analyzed. The samples were stored at -80 °C for a period of 46 to 150 days (mean 115.9 days, SD 44.3 days).

### AT-derived stem cells harvested by TEC or ED were viable and expanded successfully in vitro

**Fig. 1:**
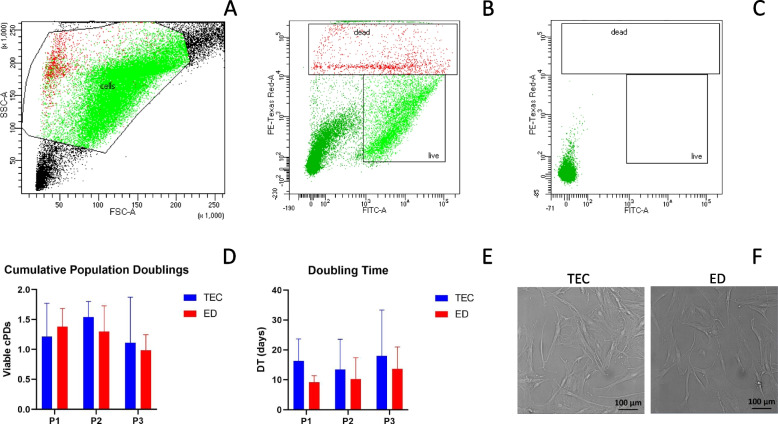
Viability and cell culture. Flow cytometry data of a LIVE/DEAD® Viability/Cytotoxicity Kit for mammalian cells. (**A**) Gating of stem cells based on side scatter (SSC-A) and forward scatter (FSC-A) to exclude debris and leucocytes, and (**B**) Sample staining showing gating on live and dead cells based on green and red fluorescence following isolation with enzymatic digestion (ED) immediately after thawing. (**C**) Unstained control showing no false positive on the flow cytometry viability assay. Box plot showing (**D**) viable cumulative population doublings (cPDs) and (**E**) doubling time (DT) (in days) of stem cells at passage (P) 1 to 3 from cryopreserved microfragmented adipose tissue (AT) (*n* = 7) when isolated with tissue explant culture (TEC) and ED, respectively. Error bars indicate standard deviation. (**F**) Representative brightfield images at 20 × magnification of paired monolayer cells at passage 4 when isolated with TEC and ED, respectively, and cultured in normal expansion medium

Viable stem cells were successfully recovered and expanded from all patients (n = 7) using both isolation methods. In 3 mL microfragmented AT when processed with ED, mean stem cell numbers were 394,917 (SD 201,864) with a mean viability of 81.06% (SD 3.93) immediately after thawing. Representative images of viability flow cytometry data are shown in Fig. 1. Most dead cells were more granulated (higher side scatter (SSC-A) values) and smaller in size (lower forward scatter (FSC-A) values) than the viable stem cells (Fig. 1.A). There was no statistically significant difference in viable population doublings or doubling time from passage 1 to 3 when comparing TEC to ED (passage 1; *p* = 0.9, passage 2; *p* = 0.5, passage 3; *p* = 0.7) (Fig. 1). Cells isolated with both methods had a similar spindle shaped morphological appearance with plastic adherence as shown in Fig. 1.

### AT-derived stem cells harvested by TEC or ED have heterogenous stem cell composition

**Fig. 2:**
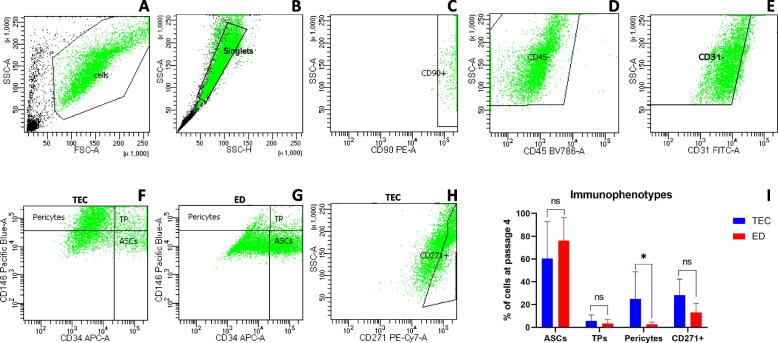
Immunophenotyping following tissue explant culture and enzymatic digestion. Gating strategy of multicolor flow cytometry. (**A**) Selection of cells of interest and removal of debris based on side scatter area (SSC-A) and forward scatter area (FSC-A), (**B**) Singlets cell selection based on SSC-A and side scatter height (SSC-H), (**C**) Selection of CD90^+^ cells, (**D**) Selection of CD45^−^ cells, (**E**) Selection of CD31^−^ cells. (**F**) Representative dot plot following tissue explant culture (TEC). Pericytes were identified as CD31^−^CD34^−^CD45^−^CD90^+^CD146^+^, transitional pericytes (TP) as CD31^−^CD34^+^CD45^−^CD90^+^CD146^+^, and adventitial stem cells (ASCs) as CD31^−^CD34^+^CD45^−^CD90^+^CD146^−^. (**G**) Representative dot plot following enzymatic digestion (ED). Subtypes were identified as stated above. (**H**) Selection of CD271^+^ cells identified as CD31^−^CD45^−^CD90^+^CD271^+^. Gating was performed based on non-stained cells, fluorescence-minus-one (FMO) and mouse IgG1κ isotype controls for each fluorophore. (**I**) Box plot showing the distribution (%) of immunophenotypes of ASCs, TPs, pericytes, and CD271^+^ stem cells from cryopreserved microfragmented adipose tissue following isolation by TEC and ED, respectively, when analyzed by flow cytometry at passage 4. The data is shown as mean values with standard deviation. ns: non-significant, *: statistically significant *p*-value < 0.05 when analyzed with paired t-tests

Multiple stem cell immunophenotypes were identified by multicolor flow cytometry in cryopreserved microfragmented AT at passage 4 when using both isolation methods. These included: adventitial stem cells (ASCs) (CD31^−^CD34^+^CD45^−^CD90^+^CD146^−^), pericytes (CD31^−^CD34^−^CD45^−^CD90^+^CD146^+^), transitional pericytes (CD31^−^CD34^+^CD45^−^CD90^+^CD146^+^), and CD271^+^ stem cells (CD31^−^CD45^−^CD90^+^CD271^+^). The distribution of immunophenotypes following TEC and ED is shown in Fig. 2. More pericytes were present when using TEC (mean 25%, SD 24%) compared to ED (mean 3%, SD 2%) (*p* = 0.04). For the presence of ASCs, no statistically significant difference was detected when using TEC (mean 60%, SD 32%) compared to ED (mean 76%, SD 20%) (*p* = 0.2). Likewise, no statistically significant difference was detected for the presence of transitional pericytes when using TEC (mean 6%, SD 5%) compared to ED (mean 3%, SD 3%) (*p* = 0.47), or for the presence of CD271^+^ stem cells when using TEC (mean 28%, SD 14%) compared to ED (mean 13%, SD 8%) (p = 0.09).

### AT-derived stem cells harvested by TEC or ED were capable of adipogenic and osteogenic differentiation in vitro

Stemness was further verified by adipogenic and osteogenic in vitro differentiation performance.

### Adipogenic differentiation: Oil-Red-O staining

**Fig. 3:**
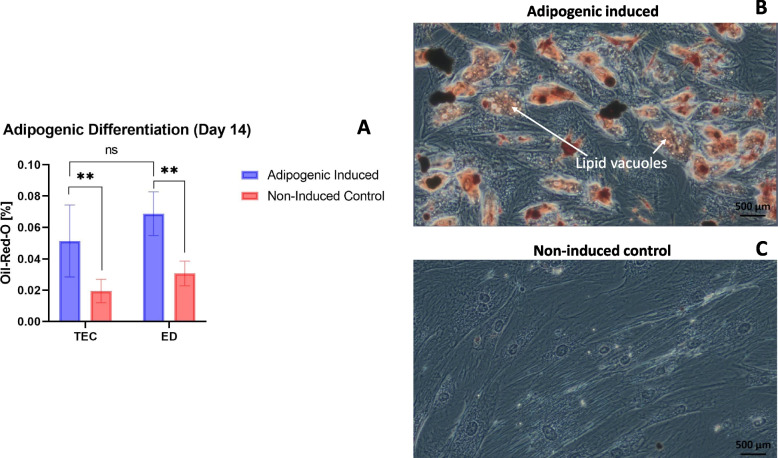
Oil-Red-O staining of adipogenic induced and non-induced cells. (**A**) Box plot of Oil-Red-O concentration (%) of adipogenic induced and non-induced paired stem cells from cryopreserved microfragmented adipose tissue when isolated with tissue explant culture (TEC) and enzymatic digestion (ED), respectively. ns: non-significant, **: *p* < 0.01 when analyzed with paired t-tests. (**B)** Phase contrast image (20x) of stem cells from cryopreserved microfragmented adipose tissue when isolated with TEC and stained with Oil-Red-O after 14 days of culture in adipogenic induction medium. Red color shows lipid vacuoles (arrows). (**C**) Phase contrast image (20x) of stem cells from cryopreserved microfragmented adipose tissue when isolated with TEC and stained with Oil-Red-O after 14 days of culture in normal expansion medium (negative control). Size bars show 500 µm

All samples were able to undergo adipogenic differentiation after 14 days of culture in adipogenic induction medium as shown in Fig. 3. Oil-Red-O concentration was statistically significant higher in adipogenic-induced cultures compared to non-induced controls when using both TEC (*p* = 0.005) and ED (*p* = 0.002), showing that all samples had adipogenic differentiation capacity. No statistically significant difference in Oil-Red-O concentration was identified for adipogenic-induced cultures when comparing TEC to ED (*p* = 0.2).

### Osteogenic differentiation: Alizarin Red S staining

**Fig. 4:**
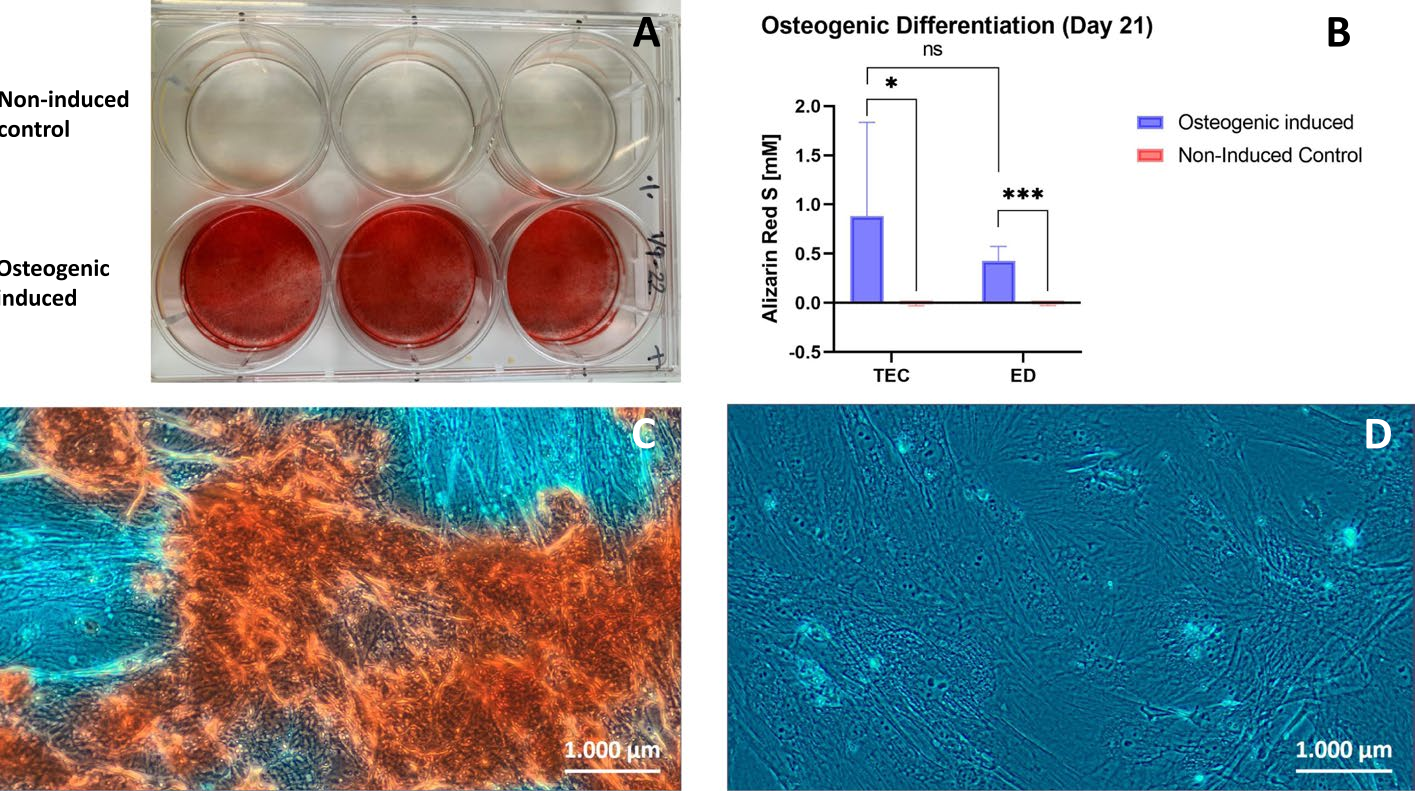
Alizarin Red S staining of osteogenic induced and non-induced cells. (**A**) Representative image of monolayer stem cells from cryopreserved microfragmented adipose tissue when isolated by tissue explant culture and cultured in osteogenic induction medium or normal expansion medium (negative control) in 6-wells for 21 days and stained with Alizarin Red S. (**B**) Box plot of Alizarin Red S concentration [mM] of osteogenic induced and non-induced paired stem cells from cryopreserved microfragmented adipose tissue when isolated with tissue explant culture (TEC) and enzymatic digestion (ED), respectively. ns: non-significant, *: *p* < 0.05, ***: *p* < 0.001 when analyzed with paired t-tests. (**C**) Phase contrast image (20x) of stem cells from cryopreserved microfragmented adipose tissue when isolated with TEC and stained with Alizarin Red S after 21 days of culture in osteogenic induction medium. Red color shows calcium deposits, and (**D**) after 21 days of culture in normal expansion medium (negative control). Size bars show 1000 µm

All samples showed osteogenic differentiation performance after 21 days of culture in osteogenic induction medium as shown in Fig. 4. ARS concentration was statistically significant higher in osteogenic-induced cultures compared to non-induced controls when using both TEC (*p* = 0.04) and ED (*p* = 0.0002), which shows that all samples had osteogenic differentiation capacity. No statistically significant difference in ARS concentration was identified for osteogenic-induced cultures when comparing TEC to ED (*p* = 0.2).

### AT-derived stem cells harvested by TEC and ED showed signs of low levels of cellular senescence after serial passages

**Fig. 5:**
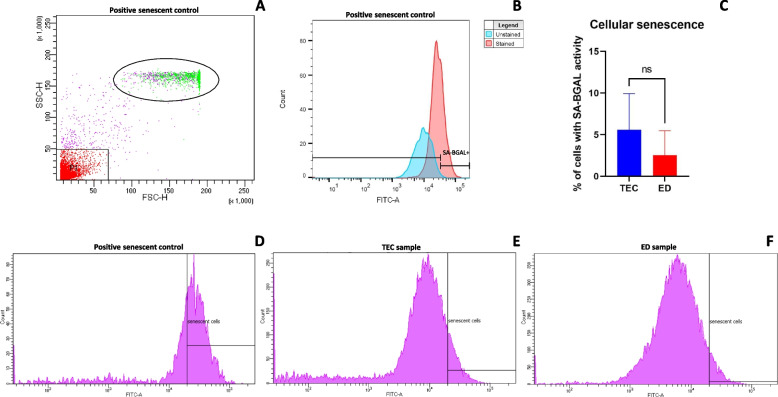
Cellular senescence evaluated by senescence associated-β-galactosidase (SA-BGAL) activity. SA-BGAL activity of cryopreserved microfragmented adipose tissue (AT) when analyzed quantitatively using flow cytometry at passage 4. (**A**) Forward (FSC-A)- and side-scatter (SSC-A) plot of senescent positive control sample (see Methods). P1 gating was used to remove debris and gate on non-P1 cells. Green colored cells (marked by ellipse) show backgating of identified senescent cells of large size and high granularity. (**B**) FITC histogram of unstained and stained positive control senescent cells used for SA-BGAL activity gating. (**C**) Box plot of the quantity (%) of cells with SA-BGAL activity from cryopreserved microfragmented AT when isolated with tissue explant culture (TEC) or enzymatic digestion (ED). ns: non-significant when analyzed with a paired t-test. (**D**) Histogram showing gating of SA-BGAL + cells as a measure of senescence from the senescence positive control. (**E**) Representative histogram showing SA-BGAL + cells as a measure of cellular senescence from cryopreserved microfragmented AT when isolated by TEC, and (**F**) when isolated by ED

Low levels of SA-BGAL activity were detected in AT-derived stem cells for both methods at passage 4, which indicates low cellular senescence. When using TEC isolation, there was a mean SA-BGAL activity in 5.6% of the cells (SD 4.3%). For ED, there was a mean SA-BGAL activity in 2.5% of the cells (SD 3.0%). No statistically significant difference was detected in SA-BGAL activity when comparing TEC to ED (*p* = 0.17) (Fig. 5). For the positive senescent control, serially cultured to undergo replicative senescence, mean SA-BGAL activity was detected in 63% of the cells. Backgating of the identified senescent cells exhibited big cell size with high granulation when using the BD FACSDiva Software 8.0.3 (Fig. 5.A – green colored cells).

## Discussion

Results from the present study shows that viable stem cells can be successfully recovered and expanded from cryopreserved microfragmented AT using both the TEC and ED isolation method after storage in 10% DMSO cryoprotective medium, which is comparable to previous limited reports on cryopreserved whole lipoaspirates and its derived stem cells [17–19]. The cell numbers and cellular viability of the present study corresponds relatively well with a previous whole AT study by Devitt et al*.* (2015) who demonstrated 4.06 × 10^4^ ± 1.36 × 10^4^ cells/g tissue with a viability of 76.05 ± 18.36% following < 1 year of cryopreservation at -70 °C [18], even though the present study used microfragmented AT and a volume instead of weight. As no standardized seeding density was used following isolation by ED, but instead all cells from a standardized volume, this may influence the days needed to reach 70% confluence at P0 for ED.

A study by Choudhery et al*.* (2014) reported that whole lipoaspirates (*n* = 10) cryopreserved for at least 1 week showed no change in spindle-shaped morphology, phenotypic markers, proliferative performance, or adipogenic-, osteogenic-, and chondrogenic differentiation potential, although the exact cryopreservation times were not mentioned [17]. In addition, it has been demonstrated that human AT-derived stem cells isolated from whole lipoaspirates (*n* = 18) retained their proliferative capacity, cell surface markers, and differentiation potential after 6 months of cryopreservation when compared to freshly isolated AT-derived stem cells [19]. For the present study, a paired comparison with data from freshly isolated AT-derived stem cells without cryopreservation was not made. The research question was instead focused on comparing two different isolation methods post cryopreservation. However, viability, cell surface markers, differentiation capacity, and cellular senescence of the current study were comparable to previous reports on fresh microfragmented AT [22, 28].

For microfragmented AT, Bianchi et al*.* (2013) reported that AT-derived stem cells could be expanded after a 10-day cryopreservation, but details concerning the study population, method, and results were lacking [9]. Importantly, the method used to (presumably) remove cell toxic cryoprotectant, DMSO, after thawing was not described, which may influence the recovery, cellular growth, and molecular structure of the cells as previously described [29, 30]. During cryopreservation, substantial cell survival and preservation of cellular integrity is, however, dependent on cryoprotective reagents, such as DMSO, to prevent bursting of cells due to ice formation, changes in osmotic pressure, and damaging solutes [31]. The findings of the current study thus add valuable information on two isolation methodologies post cryopreservation, which can be used successfully, where DMSO is rinsed out during the isolation protocols.

The present study showed that TEC and ED both can be used effectively, but with some difference between the isolation methods. Importantly, TEC provided more clinically relevant pericytes, which have been reported to have higher osteogenic performance compared to MSCs [32]. This was partly supported by the present study, which showed a trend towards higher osteogenic differentiation performance in the pericyte-rich stem cell population after TEC isolation compared to the pericyte-low stem cell population after ED, but the difference was not statistically significant. On the other hand, there was an expected trend towards obtaining faster confluence for high cell numbers when using ED compared to TEC, especially at low passages, but again this was not statistically significant.

Selection of isolation method should thus be influenced by the purpose for isolation and expansion. For research purposes, the TEC method is believed to be more representative of treatment with microfragmented AT as no enzymes have been applied. For clinical purposes, more clinically relevant pericytes can be obtained when using TEC, but it may take longer to expand the cells to sufficient cell numbers even though differences in expansion rate was not supported statistically. Additional in vivo studies on phenotypically characterized stem cells derived from microfragmented AT will be valuable to assess the clinical relevance of the pericyte content further.

Selection of -80 °C for cryopreservation was based on a recent study [18], logistic factors, and to be able to use 50 mL conical tubes, which allowed for a larger surface of the microfragmented AT to be in contact with the cryoprotective medium as AT sediments. The present study cryopreserved the samples for 46 to 150 days but was not powered to statistically compare the duration of cryopreservation. A cryopreservation of approximately 5 months (≤ 150 days) is compatible with the estimated time needed to isolate and expand the cells to clinically relevant cell numbers, depending on the tissue volume, viable cell numbers, and proliferation rate, for a second repetitive treatment 6 months after liposuction and first stem cell therapy as described by Matas et al*.* 2019 [6].

Nevertheless, it would be interesting to analyze the stability of the cells under longer cryopreservation times as a previous long-term cryopreservation study showed that long cryopreservation (> 2 years) negatively impacts initial live AT-derived stem cell isolation compared to shorter cryopreservation (< 1 year). This effect was, however, neutralized with continued cell growth [18]. The study also reported no significant effect of patient age (26–62 years) on cell isolation, viability, or growth following cryopreservation, which shows that cryopreservation may be used successfully for biobanking of stem cells from patients of varying ages. Together this indicates that cryopreservation is an efficient procedure to biobank phenotypically stable AT-derived stem cells from lipoaspirates.

This study used standard applied in vitro stem cell products. Additional studies using human clinically good-manufacturing-practice (GMP) grade counterparts would be valuable to further translate the data into the clinic.

Aging is positively correlated with the number of senescent articular chondrocytes and osteoarthritis progression [33]. It is therefore important to consider cellular senescence as a contributing factor in the pathophysiology of osteoarthritis and in therapeutic stem cell potential [13, 34]. SA-BGAL activity is considered a gold-standard biomarker of cellular senescence associated with increased size and activity of the lysosomal compartment, which releases lipofuscin [26, 35, 36]. The flow cytometric identification of large granulated senescent cells and small granulated dead cells corresponds well with previous literature on AT-derived stem cells [37]. Cellular senescence can, however, also be identified without SA-BGAL activity, specifically in senescence pathways where lysosomal activity is not involved [36]. Thus, levels of cellular senescence in the samples may be higher than the SA-BGAL assay could detect as only 63% of the cells induced to undergo replicative senescence stained positive with the applied quantitative assay. The positive cellular senescence control was previously tested functionally for cellular proliferation capacity with a 5-ethyl-2’-deoxyuridine (EdU) fluorescence proliferation assay, which confirmed that the majority of cells had undergone replicative senescence and only 4.4% of the cells proliferated after 24 h of spiking with EdU [38]. Nevertheless, the low level of cellular senescence in cryopreserved microfragmented AT following isolation with TEC and ED corresponds well with a previous study by Ragni et al*.* (2022) who reported cellular senescence in ≤ 5% of the cells from fresh microfragmented AT prior to and after processing when using a different immunophenotyping CD235a^+^ approach [22]. Moreover, the applied quantitative flow cytometric measurement of SA-BGAL positive cells is believed to be more accurate than previously used SA-BGAL histochemistry staining visualized by light microscopy [17].

The high viability, expansion capability, low levels of cellular senescence, positive stem cell markers, and differentiation performance indicates that the stem cell portion of microfragmented AT is relatively resistant to cryopreservation when stored in 10% DMSO at -80 °C under the given conditions.

## Conclusion

Viable stem cells can be isolated and expanded from cryopreserved microfragmented AT using both TEC and ED. The TEC isolation method provides more clinically relevant pericytes than ED. For research purposes, the TEC method is believed to be more representative of treatment with microfragmented AT as no enzymes have been applied.

## Data Availability

The datasets used and/or analyzed during the current study are available from the corresponding author on reasonable request.

## Abbreviations

ASCs: Adventitial stem cells
ARS: Alizarin Red S
AT: Adipose tissue
cPDs: Cumulative population doublings
DMEM: Dulbecco’s modified Eagle’s medium
DMSO: Dimethyl-sulfoxide
dPBS: Dulbecco’s phosphate buffered saline
DT: Doubling time
ED: Enzymatic digestion
EdU: 5-Ethyl-2´-deoxyuridine
FACS: Fluorescence activated cell sorting
FBS: Fetal bovine serum
FDA: U.S. federal food and drug administration
FMO: Fluorescence-minus-one
FSC: Forward scatter
GMP: Good-manufacturing-practice
KOOS: Knee Injury and Osteoarthritis Outcome Score
MSCs: Mesenchymal stromal cells
P/S: Penicillin/Streptomycin
SA-BGAL: Senescence-associated beta-galactosidase
SD: Standard deviation
SSC: Side scatter
TEC: Tissue explant culture
TP: Transitional pericytes
VAS: Visual Analog Scale
